# Nails in Feet

**Published:** 1894-02

**Authors:** John A. Bell

**Affiliations:** Watertown, N. Y.


### NAILS IN FEET.

By John A. Bell, V. S., Watertown, N. Y.

While nails in the feet are quite common in our everyday practice, I recently had a case which was a little out of the general line. We usually have nail in the foot, but this was nails in the feet, for both hind feet were found to be punctured at one and the same time, or, at least, the injuries were found at the same time. The case was a 1,500 pound horse belonging to one of my customers. It happened while I was attending our last meeting in Buffalo, and I did not get an opportunity to see it for about a week after my return, the animal having been put in the hands of another for treatment. When I first saw the case the horse was standing in a single stall on a hard floor, having just been helped up. It seemed impossible for him to stand, so I concluded to place slings under him as soon as possible, until a box stall could be arranged, when he was placed in it with difficulty. He laid down almost immediately, and, upon examination, I found the left leg badly swollen, with an exudation from the quarters. The wound had not been properly opened, and the pus was allowed to burrow and come out of the heels. The right leg had received a little better attention, the opening being large enough to allow the pus to escape.

Symptoms.—Very nervous; not able to walk; seemingly having lost the use of muscles; loss in appetite; temperature 105½; respiration hurried; bordering on septicæmia.

Treatment.—Sedatives, to regulate temperature and nerves; tonics, alteratives and stimulants to counteract septicæmia and improve appetite. Changed position from side to side three or four times daily. Removed all detached portion of sole and frog of left foot, dressing antiseptically three times daily and applying carbolized flax-seed poultice (warm). On the right, enlarged opening; dressed wound antiseptically two or three times daily. And as there was but little pus escaping and much inflammation of pedal bone, I concluded to try cold carbolized flax-seed poultices, changing two or three times daily, as case required. Although this foot did much better than the right, I did not consider it a fair test, and merely read an account of this case hoping to bring about an interchange of ideas upon whether hot or cold applications are preferable for the treatment of laminitis or nail in foot before suppurating, as there is quite a difference of opinion on this question, even amongst us to-day.

As soon as suppuration ceased, cold bran poultices were applied until the fever was reduced; then blisters were applied.

Complications.—Injury to navicular joint of left foot. Inflamation of pedal bone, or peditis in right, small quittor on each quarter of left foot and paralysis of lips due to injury while lying in stall.

The former yielded to cold applications and blisters; the quittors with peroxide hydrogen and poulticing; the lips with nerve stimulants and mild blisters over the nerve.

